# The impact of space closure versus space opening and prosthetic rehabilitation treatment on a young person's quality of life, aesthetics, and self-esteem in hypodontia: a longitudinal prospective study

**DOI:** 10.1093/ejo/cjag003

**Published:** 2026-03-10

**Authors:** Ama Johal, Rabia Dean, Mandana Amin, Shakeel Shahdad, Ferranti Wong

**Affiliations:** Department of Oral Bioengineering, Institute of Dentistry, Queen Mary University of London, Turner Street, Whitechapel, London E1 2AD, United Kingdom; Department of Oral Bioengineering, Institute of Dentistry, Queen Mary University of London, Turner Street, Whitechapel, London E1 2AD, United Kingdom; Department of Oral Bioengineering, Institute of Dentistry, Queen Mary University of London, Turner Street, Whitechapel, London E1 2AD, United Kingdom; Department of Oral Bioengineering, Institute of Dentistry, Queen Mary University of London, Turner Street, Whitechapel, London E1 2AD, United Kingdom; Department of Oral Bioengineering, Institute of Dentistry, Queen Mary University of London, Turner Street, Whitechapel, London E1 2AD, United Kingdom

**Keywords:** hypodontia, quality of life, aesthetics

## Abstract

**Background:**

The present research study uniquely aimed to evaluate the impact of undergoing orthodontic and restorative treatment on a young person’s oral health-related quality of life (QoL), self-esteem and aesthetics in relation to the management of hypodontia.

**Materials and methods:**

A prospective longitudinal hospital-based study recruited 97 participants with hypodontia, aged 11–18 years. The following questionnaires were completed both prior (T0) and after the completion of treatment by either space closure or opening and restorative rehabilitation (T1) treatment: child perception questionnaire, Bristol condition specific questionnaire for hypodontia (BCS), child health questionnaire and the Oral Aesthetic Subjective Impact Scale. The outcome variables was the end of treatment measurements, all of which were continuous in nature. The analysis was performed using analysis of covariance.

**Results:**

A total of 26 participants were lost to follow up. At the completion of treatment (T1), 71 participants completed all four questionnaires. In this cohort, there were 31 participants in the space opening and 40 in the space closure group. For both groups, improvements were observed in both QoL, self-esteem and dental aesthetics. Whilst Overall, there was no statistically significant difference detected in a number of outcomes between the two groups differences were detected in the majority of domains of the BCS, favourable towards space closure (*P* < .03).

**Conclusions:**

Treatment in participants with a range of hypodontia severity appears to have a significant positive impact, both psychologically and in terms of aesthetics. Furthermore, with the exception of the BCS, no difference in the outcomes was detected irrespective of whether participants underwent either orthodontic space closure or space opening, with subsequent prosthetic replacement.

## Introduction

Hypodontia remains the most common dental developmental disorder, characterized by absence of one or more adult teeth, excluding the third molars, with an overall reported prevalence of 3.5%–6.5% [1, 2]. Hypodontia has also been classified into mild (one to two missing teeth), moderate (three to five missing teeth) and severe (six or more missing teeth). In terms of prevalence, severe hypodontia is reported to affect 0.14%, with the majority (83%) demonstrating the milder form [1]. A multi-disciplinary approach to care is necessary to achieve optimal treatment outcomes, due to the inherent challenging complexity of treatment planning. The patient’s needs must be solved in a functional and aesthetic manner. Thus, the combined benefits of dental specialties such as orthodontic, adult restorative, paediatric and oral and maxillofacial surgical teams optimize the possibility of delivering desirable aesthetic and functional outcomes [3].

Patients with hypodontia are typically managed, based on the severity, by either a combination of orthodontic space closure alongside minimal restorative input or orthodontic space opening for prosthetic replacement of absent teeth. Although, both therapeutic options are effective, there is unfortunately limited evidence to-date to suggest that orthodontic space closure may be favoured over prosthodontic replacement and thus the decision can be a reflection of what is judged to be optimal clinical practice or preference [4, 5]. This is best illustrated in those with congenitally missing lateral incisors, where space opening has been advocated as the preferred approach, again on the basis of a very limited evidence-base [4, 5]. More recently, there has been a reported trend away from space opening and prosthetic replacement towards space closure, which may represent a change in opinion towards prosthetic replacement options, as well as higher degrees of confidence with the emergence of the application of temporary anchorage devices to aid space closure and improved biomechanics [6].

There appear to be significant shortcomings in the literature in respect of the impact of hypodontia on quality of life (QoL). Predominately, these have focussed on the impact of untreated hypodontia and are principally limited to the application of generic scales of oral health related QoL, with little recognition of the need to utilize a condition-specific questionnaire to offer a tailored understanding into the effect of hypodontia [7–14]. More recently, Johal *et al.* [15] in the first prospective longitudinal study of its kind, applied a combination of generic and a condition-specific scales for hypodontia, along with measures of self-esteem and aesthetics. The authors demonstrated, in young people, ‘untreated’ hypodontia had a very negative effect on aesthetics, function and psychosocial well-being, with severe hypodontia showing greater negative impacts [15]. Of greater importance, has been the lack of research evaluating the impact of orthodontic treatment on a young person’s QoL, applying generic and/or condition-specific measures of QoL. A study by Abu-Awwad *et al.* [12] applied a generic oral-health related QoL measure to adult patients with hypodontia and reported higher patient satisfaction with dental aesthetics following treatment [12]. Johal *et al.* [16] in their longitudinal study of the patient journey in the management of hypodontia, demonstrated that following the completion of the orthodontic stage of treatment alone, those participants undergoing space closure (treatment completion) demonstrated significantly improved QoL in a range of psychological and aesthetic scales, understandably compared with those undergoing space opening (incomplete treatment) and prior to their restorative rehabilitation. This finding in terms of aesthetic self-perception is further supported in the literature, where fixed orthodontic treatment in routine (non-hypodontia) patients, aged between 12 and 15 years, demonstrates significant improvement [17]. However, the literature to-date has failed to evaluate the effect in participants at the stage of completion of treatment in both space closure and opening groups, following the restorative phase of treatment, in a prospective manner.

Unfortunately, once again, there is little evidence exploring the effect of orthodontic treatment in adolescent patients with hypodontia in relation to self-esteem and aesthetics. A study looking at the impact of moderate and severe hypodontia in adults, with an age and gender matched control group, found no significant difference in self-esteem between the groups [10]. In terms of aesthetic outcome considerations, the literature demonstrates that older children become increasingly unhappy with their dental appearance and consequently this becomes a primary motivator for seeking orthodontic treatment [18]. Furthermore, the lack of any evidence in relation to the impact of treatment in adolescent patients, with hypodontia, needs to be quantified.

Given the sparsity of evidence available, it is essential to determine the impact of completing the full patient journey of treatment in relation to orthodontic space closure and space opening, including restorative rehabilitation, on their QoL, self-esteem and aesthetics in adolescent patients with hypodontia. As a result, the aim of the present longitudinal study was to utilize both generic and condition-specific QoL tools to assess the impact of completing treatment in adolescent participants with hypodontia on their QoL, along with their impact on self-esteem and aesthetics, for both orthodontic space closure and space opening, with restorative rehabilitation.

## Materials and methods

This was a prospective, longitudinal hospital-based study in which participants with hypodontia were recruited from those attending a multidisciplinary clinic between June 2016 and January 2017. Participants who fulfilled the selection criteria were approached and invited to participate in the study. The inclusion criteria for this study were as follows: aged between 11 and 18 years; medically fit and well; absence of any mental incapacity; able to speak and read English and with a radiographically confirmed diagnosis of hypodontia (of at least two teeth), excluding third molars. Participants were excluded on the basis of the presence of craniofacial anomaly; facial scarring or disfigurement; previously undergone orthodontic or restorative treatment to address the hypodontia or anterior aesthetics, or if there was any untreated dental disease, including caries or periodontal disease.

Ethical approval was granted by the Regional Ethics Committee (reference number 15/LO/0294). Written informed consent was obtained from participants aged 16 years and above, whereas for those aged under 16 years, written parental consent and the child’s assent were obtained. A data collection form was completed for all participants, to collect the following information: age, gender, ethnicity, teeth missing and present, any co-existing dental features associated with hypodontia, for example, microdontia or hypoplasia. All participants prior to being enrolled in the study were seen for assessment and treatment planning on a multidisciplinary treatment clinic (MDT). The team consists of Consultants from the specialities of Orthodontics, Paediatric dentistry and adult Restorative dentistry, with all participants being jointly assessed in person, with a full set of clinical records and subsequent treatment being undertaken in the single centre under their respective and joint care. For participants under the age of 16 years, any restorative treatment required was undertaken in the Paediatric department, including any prosthodontic replacement of missing teeth with resin-bonded bridges (if appropriate). Beyond this age, all restorative care after planned orthodontic treatment for space distribution, including any prosthodontic replacement of missing teeth was assessed clinically and radiographically, in conjunction with the participant’s wishes, for suitability of osseo-integrated implants or resin-bonded bridges. Thus, the decision as to whether to open or close space was an entirely non-randomized and jointly based on the advice of the MDT and the decision of the participant and parent, after being provided with all the information regarding each option provided by the former.

The research team have previously reported on the baseline characteristics of this sample [15]. In the present study, all participants completed the set of questionnaires, described below, both prior to undertaking any treatment and following the end of treatment For those in the space closing group, this was defined as after the completion of orthodontic treatment, whereas for those participants in the space opening group, this was following orthodontic treatment and prosthetic rehabilitation.

The questionnaires used were: the child perception questionnaire (CPQ), a generic scale of QoL [19], the Bristol condition specific questionnaire for hypodontia (BCS) [14, 20], the child health questionnaire (CHQ-CF-87) [21] for measurement of self-esteem and the Oral Aesthetic Subjective Impact Scale (OASIS) [22] to assess aesthetic concerns. The present study serves to compare treatment outcomes, applying the same series of four questionnaires, following completion of the patient’s journey treatment (T1), in participants undergoing orthodontic space closure alone and those undergoing orthodontic space opening and definitive prosthetic replacement.

The CPQ contains 37 questions divided into the following four domains: oral symptoms (6 items), functional limitations (9 items), emotional well-being (9 items) and social well-being (13 items). The questionnaire was designed to establish the child's view of their dental appearance and the perceived views of their peers. It enquires as to the frequency of events experienced by the child in the previous three months, with response options as follows: never = 0; once/twice = 1; sometimes = 2; often = 3; and every day/almost every day = 4. In addition, it includes two general questions broadly phrased to assess oral health and its impact on daily life. The questionnaire demonstrates validity and reliability in its application, including the UK population [19, 22, 23].

The BCS was more specifically designed to assess the relationship between hypodontia and QoL in four domains: treatment (4 items), activities (7 items), appearance (9 items) and the reaction of other people (10 items). The questionnaire includes some supplementary questions, with categorical options (rather than Likert scales) that are not meant to be aggregated into indexes. The BCS was developed in conjunction with Royal Devon and Exeter NHS Foundation Trust and Bristol University [20]. Akram *et al.* [14] have subsequently demonstrated the questionnaires validity and reliability in the assessment of QoL in adolescents participants with hypodontia.

The CHQ-CF-87 is a generic paediatric health-related QoL measure that consists of 87 questions to assess the physical and psychosocial experiences of children, aged 11–17 years [21]. A subscale of this questionnaire, consisting of 14 questions was used to assess how missing teeth affected the child's self-feeling and value in their social confidence, school activities and self-regard. The measure applies a 5-point Likert scale ranging from very good to very badly. The higher the score, the higher the self-esteem. This subscale questionnaire has been shown to be both a valid and reliable measurement tool for assessing self-esteem in children [23, 24].

The OASIS questionnaire contains five measures, each scored on a seven-point Likert scale, designed to assess the level of concern that participants have towards their dental appearance, teasing and avoidance of smiling [22].

### Statistical analysis

A formal sample size calculation determined 65 participants were required to detect a mean difference of 8.2 in the CPQ scale, at the 5% level of significance, and with a power of 80%. The number was subsequently inflated to allow for dropouts/loss to follow-up by 30% to permit long-term follow-up of participants to the completion of planned restorative care. Thus, a minimum of 85 participants was to be recruited, with an effect size of 0.5. This was based on the study of Laing *et al.* [8] which evaluated the impact of untreated hypodontia on the oral health-related QoL, applying the CPQ scale and reported a standard deviation (SD) of 16.0.

The analysis focussed on the outcomes at the end of treatment for the two groups. The outcome variables were the end of treatment measurements, all of which were continuous in nature. The analysis was performed using analysis of covariance. The main comparison was between groups (open or close space), with the equivalent outcome measurement at the pre-treatment timepoint used as a covariate in the analyses. For each group and each timepoint, the number of participants measured are reported, along with the mean and standard deviation score. Additionally, group differences for the end of treatment measurements are presented. The mean difference in outcome between groups are presented, along with corresponding confidence intervals, and are adjusted for the pre-orthodontic measurements. The differences are calculated as outcome in the open space group, minus outcome in the close space group. *P*-values indicating the significance of the group differences are also presented in the final column. Additional analyses examined the changes in outcomes from the pre-orthodontic stage to the end of treatment in each of the two groups separately. These analyses were performed using the paired *t*-test and presented as *P*-values for the significance of the within group comparisons of the change in outcome over time for each group separately.

## Results

The participant characteristics, including gender, ethnicity, prevalence of missing teeth and the severity of hypodontia, have been previously detailed and reported [15]. A total of 97 participants with a confirmed diagnosis of hypodontia were initially recruited. Of these, 58 presented with mild and 39 with moderate-severe hypodontia and with a good distribution of anterior (*n* = 41) only, posterior (*n* = 30) only and a combination (*n* = 26) of anterior and posterior missing teeth [15]. Forty-nine participants (51%) were originally planned to have closure of space and 48 participants (49%) were planned for opening of space and subsequent prosthetic replacement(s). A total of 26 (27%) participants were lost to follow up, due to an inability to contact them for follow-up care or a lack of willingness to return to hospital due to them moving geographically away from London. At the completion of treatment, 71 participants completed all four questionnaires, with 31 participants in the space opening and 40 in the space closure group.

**Figure 1:**
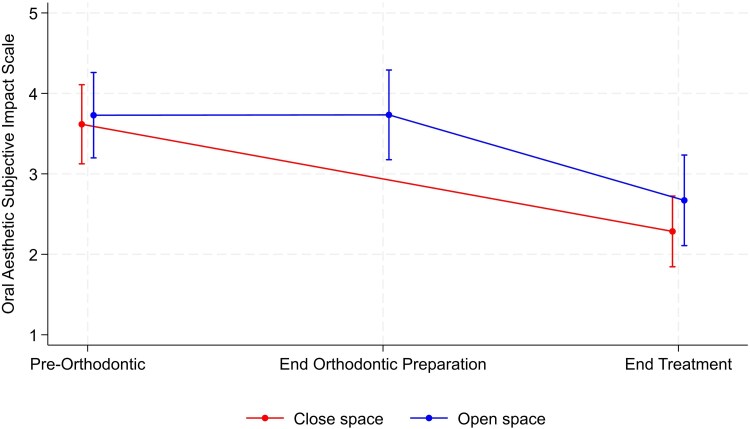
Changes in the functional limitations domain of the CPQ score, along with the corresponding confidence interval, during the hypodontia patient journey in both space opening and closure groups.

**Figure 2:**
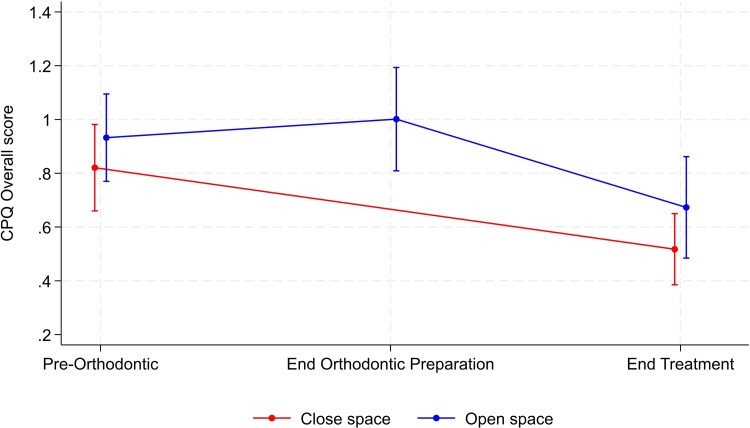
Changes in overall CPQ scores, along with the corresponding confidence interval, during the hypodontia patient journey in both space opening and closure groups.

Table 1 shows the results for the CPQ scores. The between-group comparisons suggested that there was no strong evidence of a group difference at the end of treatment for any of the subscales or for the overall score. However, there was slight evidence of a group difference for functional limitations, although this difference was only of borderline statistical significance (*P* = .05). For this outcome, there was a trend towards higher values in the open space group compared with the close space group. For the functional limitations score, values significantly decreased over time for the close space group, whilst there was no change for the open space group. The other within-group comparisons suggested some evidence of a significant change over time for both groups for the emotional well-being, social well-being and overall scores, with the values decreasing over time for these outcomes and a resultant improvement in QoL (Table 1). There was no significant change over time for the oral symptom’s outcome. Graphical illustration of the mean value at each timepoint, along with a corresponding confidence interval, over time for some of the CPQ outcomes are shown in Figs 1 and 2.

**Table 1:** The between- and within-group (open or close space) comparisons of change in the CPQ.

		Close space		Open space		Group difference	
Outcome	Timepoint	*n*	Mean ± SD	*n*	Mean ± SD	Mean (95% CI)^a^	*P*-value
Oral symptoms	Pre-orthodontic	49	1.02 ± 0.55	48	1.04 ± 0.52		
	End orthodontic preparation		—	42	1.11 ± 0.54		
	End treatment	40	0.89 ± 0.55	31	1.06 ± 0.57	0.15 (−0.11, 0.41)	.25
	Change *P*-value^b^		.15		.77		
Functional limitations	Pre-orthodontic	49	0.78 ± 0.52	48	0.92 ± 0.56		
	End orthodontic preparation		—	42	1.05 ± 0.66		
	End treatment	40	0.53 ± 0.52	31	0.81 ± 0.60	0.27 (0.00, 0.53)	.05
	Change *P*-value^b^		.02		.65		
Emotional well-being	Pre-orthodontic	49	1.10 ± 1.10	48	1.24 ± 0.98		
	End orthodontic preparation		—	42	1.32 ± 1.01		
	End treatment	40	0.57 ± 0.70	31	0.67 ± 0.76	0.09 (−0.25, 0.43)	.61
	Change *P*-value^b^		.005		.02		
Social well-being	Pre-orthodontic	49	0.57 ± 0.52	48	0.67 ± 0.60		
	End orthodontic preparation		—	42	0.70 ± 0.69		
	End treatment	40	0.31 ± 0.29	31	0.40 ± 0.47	0.09 (−0.10, 0.27)	.35
	Change *P*-value^b^		.02		.04		
Overall score	Pre-orthodontic	49	0.82 ± 0.56	48	0.93 ± 0.56		
	End orthodontic preparation		—	42	1.00 ± 0.62		
	End treatment	40	0.52 ± 0.41	31	0.67 ± 0.51	0.14 (−0.08, 0.36)	.20
	Change *P*-value^b^		.006		.06		

^a^Mean difference calculated as outcome for open space group minus outcome for close space group. Differences adjusted for equivalent measurement at pre-orthodontic phase.

^b^ *P*-value for change from pre-orthodontic phase to end of treatment phase.

**Figure 3:**
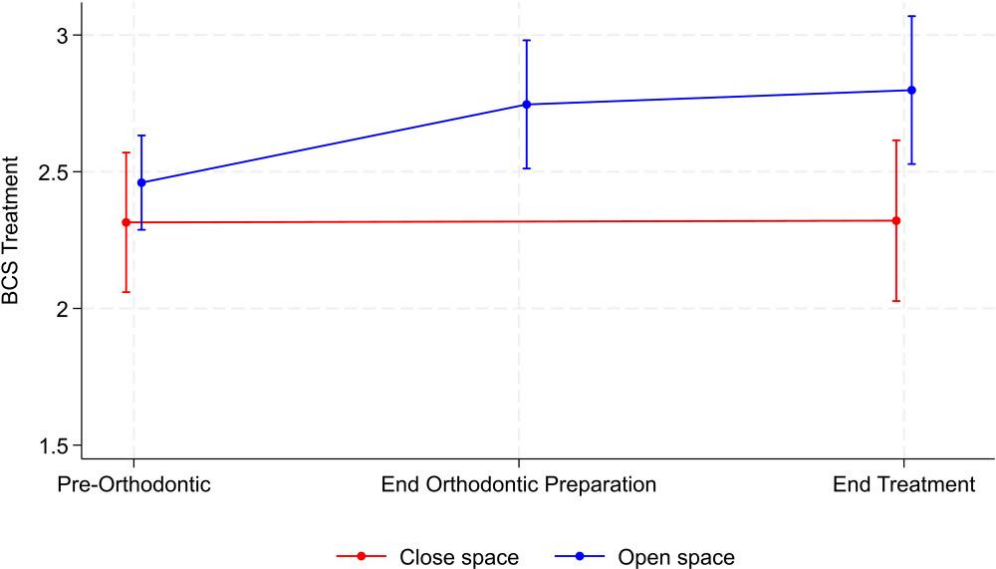
Changes in Bristol condition specific questionnaire (BCS) treatment domain scores, along with the corresponding confidence interval, during the hypodontia patient journey in both space opening and closure groups.

**Figure 4:**
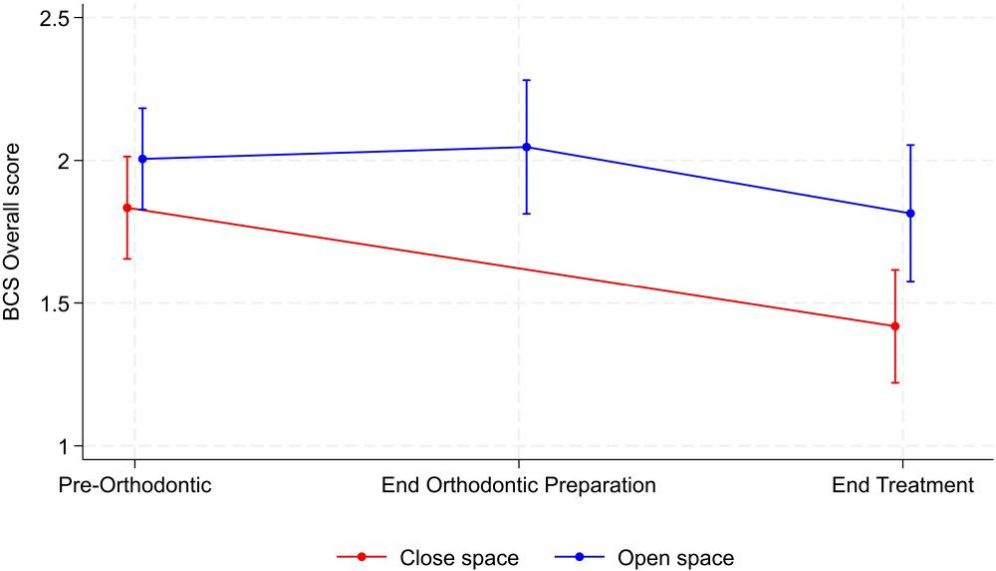
Changes in the overall Bristol condition specific questionnaire (BCS) score, along with the corresponding confidence interval, during the hypodontia patient journey in both space opening and closure groups.

Table 2 reports the results for the Bristol condition specific questionnaire (BCS) outcomes. The results suggested no evidence that the appearance domain varied between the two groups. For this scale, within-group comparison demonstrated there was a significant reduction over time in both groups, representing an improvement in this domain. However, all other domains of the questionnaire were found to be significantly different between groups, in which the scores were significantly higher, indicating greater negative impact in participants undergoing space opening with subsequent prosthetic rehabilitation compared with the space closure group. The largest difference was seen in regard to the treatment domain, where no change over time was observed in regard to the close space group, but values were almost 0.5 units higher in the open space group (Fig. 3). There was a mean difference of 0.36 units between groups for the overall score (Fig. 4). Again, within-group comparison demonstrated that for the activities and reaction of other people domains along with overall score, a significant reduction was observed over time in the space closure group but no significant change in the open space group.

**Table 2:** The between- and within-group (open or close space) comparisons of change in the Bristol condition specific questionnaire (BCS).

		Close space		Open space		Group difference	
Outcome	Timepoint	*n*	Mean ± SD	*n*	Mean ± SD	Mean (95% CI)^a^	*P*-value
Treatment	Pre-orthodontic	49	2.31 ± 0.89	48	2.46 ± 0.59		
	End orthodontic preparation		—	42	2.75 ± 0.75		
	End treatment	40	2.32 ± 0.92	31	2.80 ± 0.75	0.48 (0.08, 0.87)	.02
	Change *P*-value^b^		.70		.007		
Activities	Pre-orthodontic	49	1.40 ± 0.71	48	1.45 ± 0.79		
	End orthodontic preparation		—	42	1.68 ± 0.81		
	End treatment	40	1.06 ± 0.64	31	1.46 ± 0.65	0.38 (0.07, 0.68)	.02
	Change *P*-value^b^		.02		.97		
Appearance	Pre-orthodontic	49	2.24 ± 0.82	48	2.39 ± 0.80		
	End orthodontic preparation		—	42	2.22 ± 1.00		
	End treatment	40	1.33 ± 0.93	31	1.65 ± 0.88	0.29 (−0.14, 0.72)	.18
	Change *P*-value^b^		<.001		.004		
Reaction of other people	Pre-orthodontic	49	1.63 ± 0.72	48	1.83 ± 0.67		
	End orthodontic preparation		—	42	1.84 ± 0.82		
	End treatment	40	1.34 ± 0.70	31	1.74 ± 0.70	0.36 (0.04, 0.69)	.03
	Change *P*-value^b^		.02		.76		
Overall score	Pre-orthodontic	49	1.83 ± 0.62	48	2.01 ± 0.61		
	End orthodontic preparation		—	42	2.05 ± 0.75		
	End treatment	40	1.42 ± 0.62	31	1.81 ± 0.65	0.36 (0.07, 0.66)	.02
	Change *P*-value^b^		<.001		.34		

^a^Mean difference calculated as outcome for open space group minus outcome for close space group. Differences adjusted for equivalent measurement at pre-orthodontic phase.

^b^ *P*-value for change from pre-orthodontic phase to end of treatment phase.

**Figure 5:**
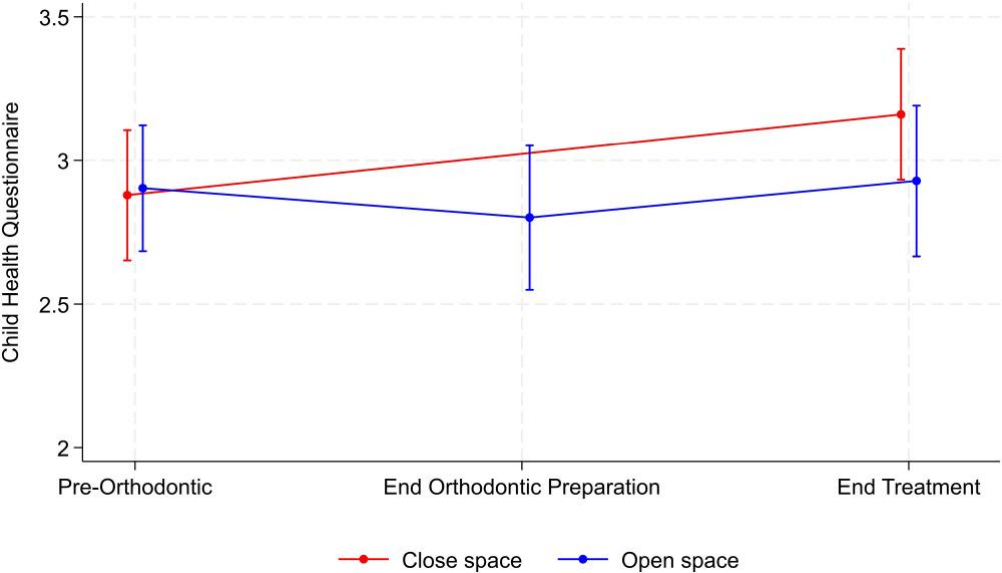
Changes in the CHQ score, along with the corresponding confidence interval, during the hypodontia patient journey in both space opening and closure groups.

**Figure 6:**
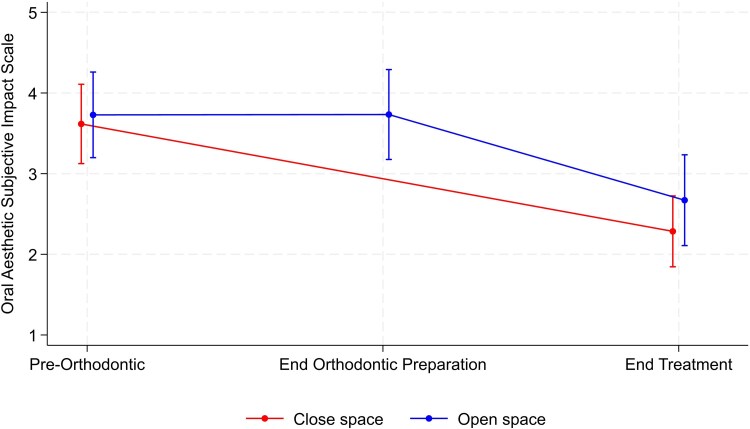
Changes in the OASIS score, along with the corresponding confidence interval, during the hypodontia patient journey in both space opening and closure groups.

The results for the CHQ for measurement of self-esteem and the OASIS are summarized in Table 3. There was no significant between-group difference at the end of treatment for either the CHQ or OASIS scores (Figs 5 and 6). Within-group comparison showed no evidence of a change over time for either group, whereas the OASIS score for both groups underwent a significant reduction from the start to the end of treatment journey, indicating improvement in perceived aesthetics (Fig. 6).

**Table 3:** The between- and within-group (open or close space) comparisons of change in the CHQ and OASIS outcome measures.

		Close space		Open space		Group difference	
Outcome	Timepoint	*n*	Mean ± SD	*n*	Mean ± SD	Mean (95% CI)^a^	*P*-value
CHQ	Pre-orthodontic	49	2.88 ± 0.80	48	2.90 ± 0.76		
	End orthodontic preparation		—	42	2.80 ± 0.81		
	End treatment	40	3.16 ± 0.72	31	2.93 ± 0.72	−0.26 (−0.59, 0.08)	.12
	Change *P*-value^b^		.07		.72		
OASIS	Pre-orthodontic	49	3.61 ± 1.71	48	3.73 ± 1.83		
	End orthodontic preparation		—	42	3.73 ± 1.79		
	End treatment	40	2.29 ± 1.37	31	2.67 ± 1.53	0.38 (−0.31, 1.06)	.27
	Change *P*-value^b^		<.001		.03		

^a^Mean difference calculated as outcome for open space group minus outcome for close space group. Differences adjusted for equivalent measurement at pre-orthodontic phase.

^b^ *P*-value for change from pre-orthodontic phase to end of treatment phase.

## Discussion

The current prospective longitudinal study is the first of its kind to comparatively evaluate the impact of the treatment journey, following either orthodontic space closure and/or space opening with restorative rehabilitation, in participants with varying degrees of hypodontia severity. This represents a key difference from the published research to-date, which has primarily focussed on the impact of treatment, often retrospectively, with inherent bias and in participants with absent maxillary lateral incisors [25–28]. Furthermore, the study uniquely assessed the impact of oral health-related QoL, using both a generic and a newly designed and validated condition-specific measure, in addition to self-esteem and aesthetics.

The studies baseline characteristics along with the severity and distribution of the hypodontia in the present sample have been previously described and analysed in detail [15]. However, given the complexity of hypodontia in the sample, it was naturally difficult to control for all the potential confounding effects observed between those who experienced hypodontia in the anterior versus posterior or a combination of both segments. In particular, the impact of these is expected to be different and most likely greater in the anterior segment, which could have been further exacerbated by the overlong retention of deciduous teeth in the posterior dentition, especially if these teeth were judged to have a good long-term prognosis, masking the impact on the participant of any otherwise potential visible gap(s). In the present study, maxillary lateral incisors were found to be the most common missing teeth, excluding the third molars, which compares favourably with the limited studies published evaluating treatment responses [25–28]. This finding needs to be interpreted with caution, as the study design does not permit an accurate estimation of prevalence. Furthermore, the literature evaluating prevalence of hypodontia has reported mandibular second premolars as being more commonly absent teeth (following third molars) [1, 2]. In contrast, the absence of anterior teeth, such as maxillary lateral incisors, is more likely to be a concern and warrant referral for treatment and therefore appear ‘more common’.

### Impact of treatment on QoL

The present study employed two different scales to assess the impact of the participant’s treatment journey, the CPQ and the BCS. Overall, in relation to the CPQ, no differences were observed in their QoL, irrespective of whether participants underwent space closure or opening. Only with the exception of the functional limitations domain, where greater reduction in scores were observed over time in favour of space closure, but this was only of borderline significance. Furthermore, when analysing the changes within each group, with the exception of the oral symptoms domain, all other domains demonstrated an improvement in QoL. In terms of the Bristol condition specific questionnaire (BCS), only the appearance domain appeared to show that there was no difference detected between the two treatment groups, with both showing a significant reduction in scores over the treatment period and therefore an improved overall impact on this domain. Thus, it is reassuring to learn that participants with hypodontia felt their appearance improved with treatment, irrespective of whether they were planned to undergo space closure or opening and prosthetic replacement. In contrast significant differences were observed in respect of all other domains of the questionnaire between the groups (space open vs. close), in which those participants undergoing space opening reported greater negative impact of their treatment on their QoL. This finding is perhaps not surprising, given that it related to the perceived treatment complexity, length and need for a false tooth, as part of their treatment journey. The remaining three domains of the BCS (activities, reaction of other people and overall score) all showed a significant reduction in scores from the commencement (pre-orthodontic point) to the completion of treatment in the space closure group, with no significant change being observed in the space opening group. These findings certainly add to our understanding of the impact of treatment in children with hypodontia and begin to address a recently acknowledged shortcoming of the current evidence-base [29]. Unfortunately, there is no published data to directly compare the current longitudinal studies findings with, in terms of hypodontia management and its impact on these two scales of QoL [29]. Abu-Awwad *et al.* [12] reported in a cross-sectional study, the impact on oral-health related QoL, using a generic scale of measure, following dental restoration in patients who had undergone space opening in hypodontia compared with reported British public norms. They reported higher average scores and therefore improved QoL in this select group of hypodontia patients following treatment, compared with the British population. Equally, there are somewhat contradictory results reported in the literature, particularly in relation to the management of developmentally absent maxillary lateral incisors. Here some have reported orthodontic space closure as having a potential advantage over prosthodontic rehabilitation, with patients reporting to be more satisfaction with their appearance [6, 25, 26], whilst others have shown space opening, followed by implant replacement to produce similar well-accepted results [27, 28]. However, all these studies have significant design limitations and as such, must be interpreted with caution, they are all retrospective in design, limited to hypodontia of the missing upper lateral incisors only, with relatively small sample sizes and consequently present a high risk of bias.

### Impact of treatment on self-esteem and dental appearance

In addition to prospectively assessing the impact of treatment in participants with hypodontia, undergoing either space opening or closure, on QoL, the present study also evaluated self-esteem (CHQ) and dental aesthetics (OASIS). In terms of self-esteem, no differences were detected between the groups, however both groups demonstrated a trend towards an improved self-esteem over the course of their treatment. Again, unfortunately there are no studies published assessing the impact on self-esteem of the completed patient treatment journey in hypodontia. It is also fair to say that the pre-treatment scores reflected a relatively high level of self-esteem and may explain the lack of any overall significant improvement with treatment [15]. In terms of the perceived dental appearance, no significant between-group difference was observed at the end of treatment. It is also worth noting that in the current sample the baseline OASIS score was higher than reported by Mandall *et al.* [22], suggesting that the current study participants with a range of hypodontia severity reported greater impact than those seeking more routine orthodontic treatment [15]. In this context, it was notable that the OASIS scores for both groups underwent a significant reduction from the start to the end of their treatment journey, indicating the positive perceived benefit of treatment in hypodontia. These findings are supported by those of a large UK-based retrospective study of 451 patients (aged 4–28 years) with hypodontia, in whom the most expressed concern related to dental aesthetics [30]. Similarly, a later qualitative study of adolescents, with hypodontia, revealed that their primary motivator for seeking treatment was for aesthetic benefits along with a desire to improve self-confidence [18].

### Strengths and limitations of the study

The present study attempted to address a number of shortcomings identified in the current literature to-date [29]. In particular, an attempt was made to include a broad range of outcome scales. QoL has frequently been assessed applying the CPQ but this is not condition-specific to hypodontia, in which some domains may have lacked relevance. In contrast, the BCS is condition-specific to those with hypodontia and therefore potentially more meaningful. In applying these two scales, reliability was assessed and reported previously, using Cronbach’s *α* applying an average score for each item and the overall indices for both CPQ and BCS [15, 16]. This not only permitted for contextualization of the use of different measures within QoL and for those participants with hypodontia, but also their application will permit a reduction in study heterogeneity, for instance within meta-analysis. Aside from the initial reporting of this study, there has been no previous studies using CHQ to assess the self-esteem in hypodontia patient [15, 16]. The OASIS index score, looked to quantify the degree of concern that the cohort had in relation to their dental appearance. Whilst a significant improvement was reported in relation to appearance, in both space closing and opening groups, Mandall *et al*. [22] study had a lower reported mean than seen in the present study but assessed all types of malocclusion representative of the general population. However, the present study, specifically recruited participants with varying levels of hypodontia severity, attending a hospital-setting, which can affect their dental appearance concerns.

The current study is not without its limitations and these need to be acknowledged. A total of 26 participants were lost to follow up, which constitutes 27%. However, given the prospective and longitudinal nature of this study and the fact that no such study has been attempted or reported in patients with the full range of hypodontia severity and through to the completion of their treatment journey, it is somewhat unique and understandable. Furthermore, this was below the anticipated dropout/loss to follow-up value, allowed for in the sample size estimation. Seventy-one participants completed all four questionnaires, of which 31 participants where in the space opening and 40 in the space closure group. The challenges of undertaking such a prospective longitudinal study have been acknowledged by Silveira *et al*. [26], in their systematic review of prosthetic replacement versus space closure. The authors concluded that whilst prospective studies were required to provide more compelling scientific evidence, they equally presented difficulties and limitations on the investigation of this subject [26]. Furthermore, the findings of the current study may not be generalizable, given the study was performed in a single-hospital setting. However, such studies are inherently challenging to conduct across a broader geographic base and thus we opted to focus the study at a single centre recognized for its expertise in the field to offering the optimal multi-disciplinary environment for participant care.

## Conclusion

Treatment in participants with a range of hypodontia severity appears to have a significant positive impact, both psychologically and in terms of aesthetics.

No differences were detected in self-esteem or dental appearance outcomes when participants undergo either orthodontic space closure or space opening, with subsequent prosthetic rehabilitation.

Differences were detected in a range of domains within a condition-specific measure of QoL between participants undergoing space closure and those undergoing space opening, with subsequent prosthetic rehabilitation.

Clinicians involved in the management of hypodontia need to be aware of these implications, to facilitate shared-decision making with patients and informed consent.

## Data Availability

The datasets used and/or analysed during the current study are available from the corresponding author, upon reasonable request.
